# Management of two extremely aggressive signet ring cell colon carcinoma: a case series

**DOI:** 10.1093/jscr/rjag494

**Published:** 2026-06-23

**Authors:** Nazila Dalir, Glenn S Parker, Rachel Amanda Masia, Patricia Torres

**Affiliations:** Department of Surgery, Jersey Shore University Medical Center, 1945 NJ-33, Neptune City, NJ 07753, United States; St. George’s University School of Medicine, St. George’s, Grenada, West Indies; Department of Surgery, Jersey Shore University Medical Center, 1945 NJ-33, Neptune City, NJ 07753, United States; Department of Surgery, Jersey Shore University Medical Center, 1945 NJ-33, Neptune City, NJ 07753, United States; Department of Surgery, Jersey Shore University Medical Center, 1945 NJ-33, Neptune City, NJ 07753, United States

**Keywords:** signet ring cell carcinoma, colorectal cancer, case series, microsatellite instability, peritoneal metastasis

## Abstract

Signet ring cell carcinoma (SRCC) of the colon is a rare histologic subtype of colorectal cancer, accounting for <1% of cases, and is associated with aggressive clinical behavior and poor prognosis. We report two cases with advanced colonic SRCC. The first case involved a 58-year-old female with obstructing rectal SRCC and extensive pelvic invasion with adnexal metastasis. The second is a 45-year-old male with an obstructing splenic flexure SRCC and peritoneal dissemination, found to have microsatellite instability and multiple oncogenic alterations. Both cases required aggressive surgical intervention and illustrate the need for individualized, multimodal treatment strategies. Colonic SRCC is an uncommon but highly aggressive malignancy that frequently presents at an advanced stage. Early recognition, comprehensive pathologic and molecular evaluation, and coordinated multimodal management are critical to optimizing outcomes.

## Introduction

Primary signet ring cell carcinoma (SRCC) of the colon is a rare histologic subtype of colorectal adenocarcinoma characterized by malignant cells containing abundant intracytoplasmic mucin that displaces the nucleus peripherally, producing a signet ring appearance [1, 2]. SRCC is diagnosed when signet ring cells comprise >50% of the tumor cell population. Although more commonly associated with gastric cancer, primary colorectal SRCC accounts for ~0.6%–1.0% of colorectal malignancies and demonstrates distinct clinical and biological behavior compared with conventional adenocarcinoma [2–4].

The pathogenesis of colorectal SRCC remains incompletely defined but is believed to differ from the classic adenocarcinoma sequence. Proposed mechanisms include early loss of cell adhesion, dysregulated mucin production, and enhanced epithelial-mesenchymal transition, contributing to diffuse infiltrative growth and aggressive behavior. Molecularly, SRCC exhibits heterogeneity, with many tumors demonstrating microsatellite stability and TP53 alterations, while a subset displays microsatellite instability or aberrant Wnt/β-catenin signaling. These features may underlie the tendency for early lymphovascular invasion, peritoneal dissemination, and discordantly low serum carcinoembryonic antigen (CEA) levels despite advanced disease.

Clinically, colorectal SRCC is associated with younger age at diagnosis, advance stage at presentation, and poor prognosis. Population-based studies report that > 80% of patients present with stage III or IV disease, with 5-year overall survival rates of ~25%–30%, significantly worse than those observed in conventional colorectal adenocarcinoma [2–4]. Owing to its rarity and aggressive biology, SRCC remains underrepresented in prospective trials, and optimal management strategies are not well defined, necessitating continued reporting of detailed clinicopathologic cases.

## Case 1

A 58-year-old female presented with progressive constipation, rectal pain, and intermittent rectal bleeding over several months. Colonoscopy demonstrated a circumferential, obstructing rectal mass ~15 cm from the anal verge, preventing passage of the scope. Contrast-enhanced computed tomography (CT) of the abdomen and pelvis revealed a large rectal mass with extensive regional lymphadenopathy, invasion into adjacent pelvic organs, including the uterus, and a heterogenous left adnexal cystic lesion, suspicious for metastatic involvement (Fig. 1).

**Figure 1:**
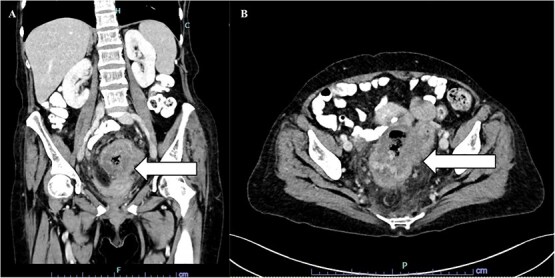
CT of the abdomen and pelvis: a large distal sigmoid/rectal mass with areas of central necrosis (coronal (A) and axial (B) views).

Serum CEA was <2 ng/ml, consistent with reports describing discordant low CEA levels in SRCC despite significant tumor burden [3, 5]. Staging laparotomy confirmed locally advanced disease with extensive pelvic involvement. The patient underwent exploratory laparotomy, Hartmann’s procedure with end colostomy, partial proctectomy, left salpingo-oopherectomy, and radical hysterectomy.

Final pathology demonstrated poorly differentiated, SRCC (poorly cohesive, carcinoma, grade 3) with patchy neuroendocrine differentiation. Immunohistochemistry was positive for CK20 and CDX2, supporting colorectal origin, and demonstrated P53 overexpression; PAX8 and estrogen receptor were negative, excluding a primary gynecologic cause [6]. Patchy synaptophysin and chromogranin, which our patient expressed, have been previously described in poorly differentiated colorectal carcinomas with mixed features [7]. Mismatch repair proteins were intact, indicating microsatellite stability. PD-L1 expression was low (tumor proportion score <1%), consistent with prior observations that many SRCCs are microsatellite stable, and immunologically ‘cold’ tumors [8, 9].

Pathologic staging revealed pT4a disease with extensive nodal involvement (pN2b; 26/34 lymph nodes positive) and metastatic involvement of the left adnexa, consistent with pM1b disease. The patient recovered uneventfully and is scheduled to initiate systemic chemotherapy.

## Case 2

A 45-year-old male presented with several weeks of diarrhea, lower gastrointestinal bleeding, unintentional weight loss, and crampy abdominal pain. CT imaging revealed a 14 cm obstructing mass centered at the splenic flexure. Flexible sigmoidoscopy confirmed a completely obstructing malignant-appearing lesion ~60 cm from the anal verge (Fig. 2).

**Figure 2:**
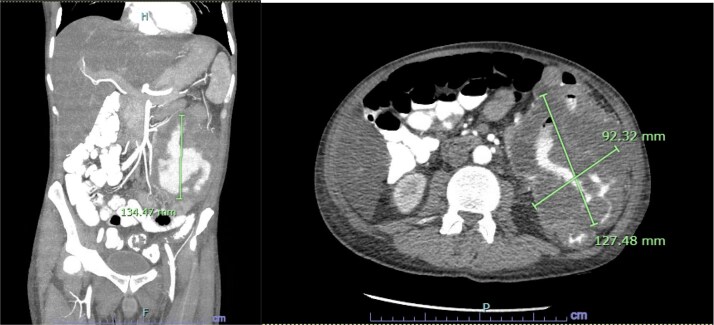
CT of the abdomen and pelvis: a large mass at the splenic flexure measuring ~13 × 9 × 13 cm (coronal and axial views, respectively).

Serum CEA was mildly elevated at 5.9 ng/ml. He reported heavy alcohol use, former tobacco use, and current marijuana use. Family history was significant for colon cancer, consistent with prior reports noting a higher prevalence of familiar risk factors in younger SRCC patients [2, 4].

The patient underwent exploratory laparotomy with low anterior resection, left hemicolectomy, and small bowel resection for contiguous tumor involvement (Fig. 3). Histopathologic examination demonstrated poorly differentiated adenocarcinoma with prominent signet ring cell features. Tumor cells stained positive for mucin, CK20, and CDX2, confirming colorectal origin [6].

**Figure 3:**
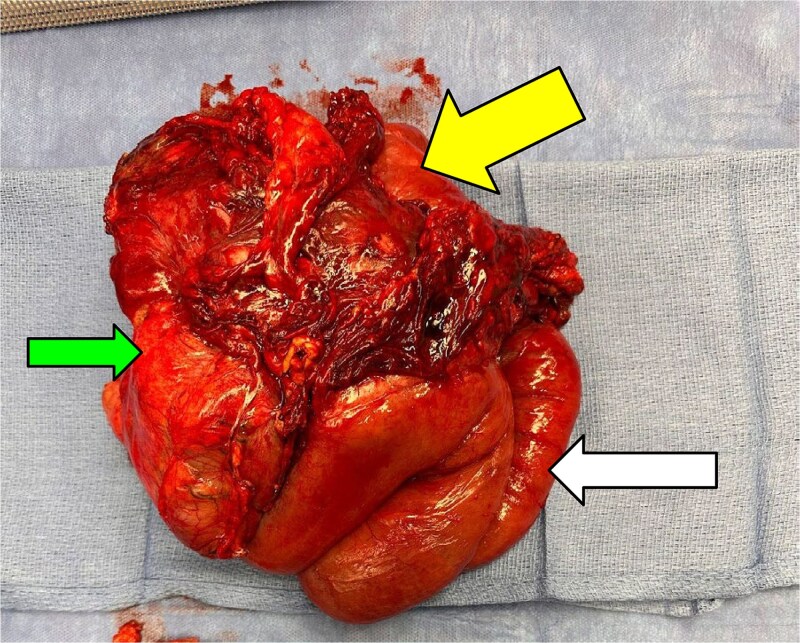
A macroscopic view of the resected specimen. Green arrow represents colon, yellow arrow represents necrotic, perforated mass, and white arrow represents small bowel.

Final staging was pT4b, pN1a (1/13 lymph nodes positive) with peritoneal involvement consistent with pM1c disease. Mismatch repair testing demonstrated loss of MLH1 and PMS2, consistent with microsatellite instability, a molecular subtype reported in a minority of SRCCs but associated with potential responsiveness to immune checkpoint inhibition [8, 9]. Molecular profiling identified mutations in TP53, ERBB2, ERBB3, CTNNB1, RNF43, and SETD2. The absence of RAS and RAF mutations has been associated with potential sensitivity to anti-EGFR-directed therapy in colorectal cancer [10]. Markedly elevated MYC and Ki-67 expression supported a highly proliferative phenotype, consistent with aggressive tumor biology [11].

## Discussion

Colorectal SRCC is consistently associated with advanced-stage presentation and inferior survival compared with conventional adenocarcinoma [3, 4, 12]. Population-based analysis from SEER and NCDB datasets demonstrate that over 80% of patients with SRCC present with stage III or IV disease and reported 5-year overall survival ranges from ~25% to 30%, compared with 50% to 60% for non-signet colorectal adenocarcinoma [3, 4, 12]. Signet ring histology has been identified as an independent adverse prognostic factor, even after adjustment for stage, grade, and treatment [4, 12].

The cases presented here illustrate classic patterns described in the literature, including extensive local invasion, early metastatic spread, and low or discordant CEA levels [3, 5]. Case 1 highlights the tendency for contiguous pelvic organ invasion and adnexal metastasis, a phenomenon attributed to diffuse infiltrative growth and transcoelomic spread [4, 8]. Case 2 demonstrates peritoneal dissemination (pM1c), a metastatic pattern that is disproportionately common in SRCC and is associated with particularly poor outcomes [8, 13].

Management of SRCC requires a multimodal approach. Surgical resection is frequently necessary for obstruction, bleeding, or local disease control, but systemic disease ultimately drives prognosis. Retrospective series evaluating cytoreductive surgery with or without hyperthermic intraperitoneal chemotherapy (HIPEC) have consistently identified signet ring histology as a negative prognostic factor, with reported median overall survival of ~12–15 months and 5-year survival <15% in patients with peritoneal metastasis [8, 13]. Nonetheless, long-term survivors have been reported in highly selected patients with limited peritoneal burden and complete cytoreduction [8].

Systemic therapy decisions should be guided by comprehensive molecular profiling. While many SRCCs are microsatellite stable and derive limited benefit from immunotherapy, MSI-high tumors may respond favorably to immune checkpoint inhibitors [9]. Additionally, identification of actionable alterations, such as ERBB2 amplification or RAS/RAF wild-type status, may inform targeted treatment strategies in selected patients [10]. Despite these advances, outcomes remain poor, emphasizing the need for further research and inclusion of SRCC patients in prospective clinical trials.

## Conclusion

Primary colonic SRCC is a rare but highly aggressive malignancy associated with advanced presentation and poor prognosis. These cases underscore the importance of early diagnosis, detailed histopathologic and molecular evaluation, and coordinated multimodal management. Improved understanding of SRCC biology and expanded access to targeted and immunotherapeutic approaches are essential to improving outcomes in this challenging disease.
